# Serum Palmitoleic Acid and Arachidonic Acid as a Noninvasive Screening Tool for Endometrial Cancer

**DOI:** 10.3390/cancers18132133

**Published:** 2026-07-01

**Authors:** Nagi Yamazaki, Yuki Katoh, Akiko Kubo, Masaki Sugawara, Yuichiro Otsuka, Tadashi Ogawa, Mamiko Fukuta, Koji Suzuki, Kenji Wakai, Yosuke Fujii, Shuichi Hirai

**Affiliations:** 1 Division of Anatomical Science, Department of Functional Morphology, Nihon University School of Medicine, 30-1 Ohyaguchi-Kami-Cho, Itabashi-ku, Tokyo 173-8610, Japan; mena24120@g.nihon-u.ac.jp (N.Y.); fujii.yosuke@nihon-u.ac.jp (Y.F.); hirai.shuichi@nihon-u.ac.jp (S.H.); 2 Department of Medicine, Nihon University School of Medicine, 30-1 Ohyaguchi-Kami-Cho, Itabashi-ku, Tokyo 173-8610, Japan; 3 Department of Obstetrics and Gynecology, Keio University School of Medicine, 35 Shinano-machi, Shinjuku-ku, Tokyo 160-8582, Japan; masaki.sugawara@keio.jp; 4 Division of Dermatology, Department of Internal Related, Kobe University Graduate School of Medicine, 7-5-1 Kusunoki-Cho, Chuo-ku, Kobe 650-0017, Hyogo, Japan; yuba@med.kobe-u.ac.jp; 5 Division of Public Health, Department of Social Medicine, Nihon University School of Medicine, 30-1 Ohyaguchikami-cho, Itabashi-ku, Tokyo 173-8610, Japan; otsuka.yuichiro@nihon-u.ac.jp; 6 Department of Legal Medicine, Aichi Medical University School of Medicine, 1-1 Yazakokarimata, Nagakute 480-1195, Aichi, Japan; ogawatd@aichi-med-u.ac.jp (T.O.); mfukuta@aichi-med-u.ac.jp (M.F.); 7 Department of Preventive Medical Sciences, Fujita Health University School of Medical Sciences, 1-98 Dengakugakubo, Kutsukake-cho, Toyoake 470-1192, Aichi, Japan; ksuzuki@fujita-hu.ac.jp; 8 Department of Preventive Medicine, Nagoya University Graduate School of Medicine, 65 Tsurumai-cho, Showa-ku, Nagoya 466-8550, Aichi, Japan; wakai.kenji.y2@f.mail.nagoya-u.ac.jp

**Keywords:** lipidomics, endometrial cancer, liquid biopsy, serum free fatty acids, screening, early detection

## Abstract

**Background/Objectives**: Endometrial cancer (EC) is the most prevalent gynecological malignancy in developed countries, yet no clinically validated noninvasive screening biomarker exists. Fatty acid metabolic reprogramming is a hallmark of cancer that produces measurable changes in systemic free fatty acid (FFA) profiles. We aimed to identify serum FFAs altered in EC and evaluate their potential as early diagnostic biomarkers. **Methods**: Gene expression of fatty acid metabolic enzymes in EC tissues was assessed using public databases. Serum levels of 19 FFAs were quantified by gas chromatography–mass spectrometry in 72 patients with EC and 84 healthy donors. A two-variable diagnostic model was constructed using logistic regression and validated in an independent dataset. **Results**: Multiple fatty acid metabolic enzymes involved in de novo synthesis were upregulated in EC tissues as early as stage I, while enzymes mediating fatty acid uptake and release were downregulated. Serum levels of palmitoleic acid, oleic acid and dihomo-γ-linolenic acid were significantly elevated, whereas arachidic acid and arachidonic acid were significantly decreased in patients with EC from stage I onward. A diagnostic model combining palmitoleic acid and arachidonic acid achieved an area under the receiver operating characteristic curve of 0.9674, with a sensitivity of 91.3% and specificity of 96.4% in the validation dataset, including for stage I disease, and was independent of body mass index, age, metastasis status, and family history. **Conclusions**: Serum FFA profiling represents a promising noninvasive strategy for early EC detection, with the potential to complement existing diagnostic methods.

## Simple Summary

Endometrial cancer is the most common gynecological cancer in developed countries, yet no reliable noninvasive screening method currently exists. Cancer cells alter their fat metabolism, leading to measurable changes in the types and amounts of fatty acids circulating in the blood. In this study, we analyzed blood samples from 72 patients with endometrial cancer and 84 healthy individuals and identified specific fatty acids that were consistently altered in patients with cancer, even at the earliest stages of the disease. Using these fatty acids, we developed a diagnostic model that accurately distinguished patients with cancer from healthy individuals using a simple blood test. This model performed well regardless of patient body mass index, age, metastasis status or family history. These findings suggest that measuring fatty acids in the blood may provide a practical, noninvasive approach to early endometrial cancer screening.

## 1. Introduction

Endometrial cancer (EC) is the most common gynecological malignancy in developed countries and ranks as the sixth most frequently diagnosed cancer among women worldwide [1]. The global incidence of EC has increased over the past decade, primarily driven by the rising prevalence of obesity and diabetes, population aging, and increasing use of hormone replacement therapy, with this upward trend projected to continue [2,3,4]. Although diagnoses have risen across all age groups, the increase in incidence among women under 40 years of age has been particularly pronounced, raising concerns about this disease in younger populations [5,6]. The prognosis of EC is heavily dependent on stage, with 5-year survival rates of 95% for stage I disease, declining to 17% for stage IVA disease and as low as 15% for stage IVB disease [7,8]. Therefore, the development of early screening and diagnostic technologies applicable across all age groups is crucial for reducing EC-related mortality.

Current diagnostic approaches for EC present significant limitations for early detection and screening. Transvaginal ultrasonography, though noninvasive, demonstrates limited specificity because of overlapping endometrial thickness measurements between malignant and benign conditions [9,10,11]. Cervicovaginal cytology lacks sufficient sensitivity for reliable cancer detection [12], while tissue-based sampling methods, despite being the diagnostic gold standard, are limited by their invasive nature, patient discomfort, and procedural failure rates [6,13]. To address these limitations, liquid biopsy approaches have emerged as promising alternatives, including blood-based assays analyzing circulating microRNAs [14] and metabolomic profiles [15], urine-based DNA methylation tests [16], and genomic/proteomic analyses of uterine and cervicovaginal fluids [17,18,19]. However, despite numerous investigations into various screening modalities, no clinically validated biomarker has yet been established for routine EC screening. This gap underscores the need for novel, accessible biomarkers that can effectively stratify patient risk and facilitate early detection.

Metabolic reprogramming is a hallmark of cancer, enabling tumor cells to adapt to their microenvironment and sustain proliferation [20]. Among various metabolic alterations, dysregulation of fatty acid metabolism has recently emerged as a critical feature of cancer pathogenesis and represents an important target for cancer treatment and diagnosis [21,22]. We have previously demonstrated in colorectal, lung, and cervical cancers that aberrant expression of key fatty acid metabolic enzymes, including stearoyl-CoA desaturase 1 (SCD1), induces alterations in intracellular fatty acid composition, leading to enhanced membrane fluidity and reduced endoplasmic reticulum stress, thereby promoting cell survival and metastatic potential. Importantly, these metabolic alterations manifest not only locally within the tumor but also systemically, resulting in measurable changes in serum free fatty acid (FFA) profiles [23,24].

Extending these observations, we further demonstrated in ovarian and cervical cancers that alterations in fatty acid metabolic enzyme expression occur as early as stage I and that specific serum FFAs enable highly accurate early diagnosis [25,26]. Specifically, in ovarian cancer, serum levels of oleic acid and arachidic acid established a highly accurate early diagnostic model [25], while in cervical cancer, stearic acid and dihomo-γ-linolenic acid levels distinguished malignant from benign conditions with high sensitivity and specificity from stage I [26]. These findings suggest that serum FFA profiling can serve as an effective early diagnostic approach for cancers exhibiting fatty acid metabolic reprogramming in tumor tissues.

In EC, alterations in various fatty acid metabolic enzymes, including SCD1 and fatty acid synthase (FASN), have been reported [27,28,29,30]. However, whether these alterations occur in early-stage disease and which specific serum FFAs are significantly affected remain unclear. Here, we aimed to identify FFAs that are altered in the serum of patients with EC and to evaluate the potential of serum FFA profiling as a novel early screening strategy for this disease.

## 2. Materials and Methods

### 2.1. Clinical Samples

Serum samples were obtained from 72 patients with EC and 84 healthy donors (Table 1). Serum samples from patients with EC were purchased from ProteoGenex (Inglewood, CA, USA) and were collected between 2024 and 2025. Serum samples from healthy donors were collected at Nihon University and CoBiA (Platform of Supporting Cohort Study and Biospecimen Analysis). Healthy donor serum was provided by Nihon University and CoBiA (Platform of Supporting Cohort Study and Biospecimen Analysis), and every sample was kept frozen at −80 °C prior to analysis. Serum samples from patients with EC were obtained commercially and were not collected under fasting conditions; information regarding the time of day of collection was not available. ProteoGenex also supplied the corresponding clinicopathological data, in which each EC case was assigned a stage from I to IV based on the 2018 International Federation of Gynecology and Obstetrics (FIGO) clinical staging system. For these patients, serum had been obtained consecutively from those who consented, prior to tumor resection during their initial surgery. Written informed consent had been obtained from all donors of the commercially purchased endometrial cancer serum samples by the supplier (ProteoGenex), and from all healthy donors provided by CoBiA at the time of collection. For the healthy donor samples obtained at Nihon University, the requirement for written informed consent was waived owing to the retrospective study design, with an opt-out opportunity provided instead.

### 2.2. Measurement of Serum FFAs by Gas Chromatography–Mass Spectrometry (GC-MS)

Measurement of serum FFAs by GC-MS was performed according to previous descriptions [25]. Briefly, FFAs were extracted from 20 µL of serum spiked with an internal standard (100 ng of margaric acid) using an ISOLUTE SLE+ column (Biotage, Vimpelgatan, Uppsala, Sweden) and dichloromethane, dried under nitrogen, and trimethylsilylated with BSTFA + TMCS (99:1) reagent (Thermo Fisher Scientific, Waltham, MA, USA).

Analysis was carried out on a Shimadzu (Shimadzu Corporation, Kyoto, Japan) GC–MS QP2010 Ultra with an Rtx-5MS column (30 m, 0.25 mm, 0.25 µm df) under 70 eV electron ionization, using the oven program and carrier gas conditions described previously [25]. The target ions (*m*/*z*), retention times, limits of detection, and limits of quantification for the 19 FFAs are listed in Table 2.

### 2.3. Gene Expression Analysis with Public Databases

Transcriptomic, clinical, and biospecimen data for uterine corpus endometrial carcinoma from The Cancer Genome Atlas project were obtained from the GDC Data Portal. To restrict the cohort to primary EC cases, samples with either of the following two clinical annotations were excluded: tumor_descriptor_malignancy = Recurrence, or synchronous_malignancy = Yes. A total of 575 samples, including 35 normal endometrial samples and 540 tumor samples, were analyzed.

Tumor stage was reassessed according to FIGO 2009 because the dataset included cases staged according to the FIGO 1988, FIGO 1995, and FIGO 2009 systems; five cases classified as stage IIA under FIGO 1988 were reassigned to stage I. The final cohort included 344 stage I, 45 stage II, 123 stage III, and 28 stage IV tumors.

All analyses were conducted in RStudio using R software version 4.5.0. Differential gene expression between normal and EC samples was analyzed using TPM-normalized bulk RNA-sequencing data and the Bioconductor package *limma* with the empirical Bayes method. The Benjamini–Hochberg procedure was used to adjust *p*-values to control the false discovery rate (FDR). Genes with an FDR of <0.05 and |log_2_ fold change| > 0.6 were defined as differentially expressed genes.

For heatmap analysis, mean expression values were calculated for each group, followed by gene-wise Z-score normalization across groups. Genes were hierarchically clustered using Euclidean distance and complete linkage. Heatmaps were generated using the Bioconductor package *ComplexHeatmap*.

### 2.4. Construction of Optimal Diagnostic Model for EC

To construct the optimal diagnostic model for EC, samples were first stratified by malignancy (control, early-stage cancer, and advanced cancer) and then randomly divided into a discovery set and a validation set at a 2:1 ratio. Second, to develop a parsimonious diagnostic model while accounting for metabolic dependencies, fatty acids were categorized into four functional clusters based on their biosynthetic pathways: saturated substrates, monounsaturated products of SCD1, n-6 polyunsaturated fatty acids, and n-3 polyunsaturated fatty acids. Following univariate logistic regression screening to identify potential predictors, we performed an exhaustive combinatorial search of all possible two-variable combinations. For each pair, a logistic regression model was fitted, and the area under the receiver operating characteristic curve (AUC) was calculated to evaluate predictive accuracy. The final optimal pair was selected based on two criteria: statistical performance (maximization of AUC) and biological robustness, ensuring that variables were selected from distinct metabolic clusters to minimize multicollinearity and incorporate complementary biological information. Third, the performance of the diagnostic index (DI) was assessed in the validation dataset.

### 2.5. Statistical Analysis

Group comparisons were performed with two-tailed Student’s *t*-tests, paired or unpaired as appropriate for the data structure, or with the nonparametric Mann–Whitney U test. Where more than two groups were compared, one-way analysis of variance with Tukey’s or Bonferroni’s post hoc correction was applied. For each FFA, sensitivity, specificity, and the corresponding AUC were determined. The relationship of the DI (palmitoleic acid, arachidonic acid) to body mass index (BMI) and to age was examined by Pearson’s correlation. Data handling and statistical computations were conducted in Stata 18.0 (StataCorp LLC., College Station, TX, USA), Prism 10.4.1 (GraphPad Software, La Jolla, CA, USA), and MetaboAnalyst 6.0, with statistical significance defined as *p* < 0.05 throughout. Values are expressed as the mean ± standard deviation.

## 3. Results

### 3.1. Altered Expression of Fatty Acid Metabolism-Related Enzymes in EC Tissues

**Figure 1:**
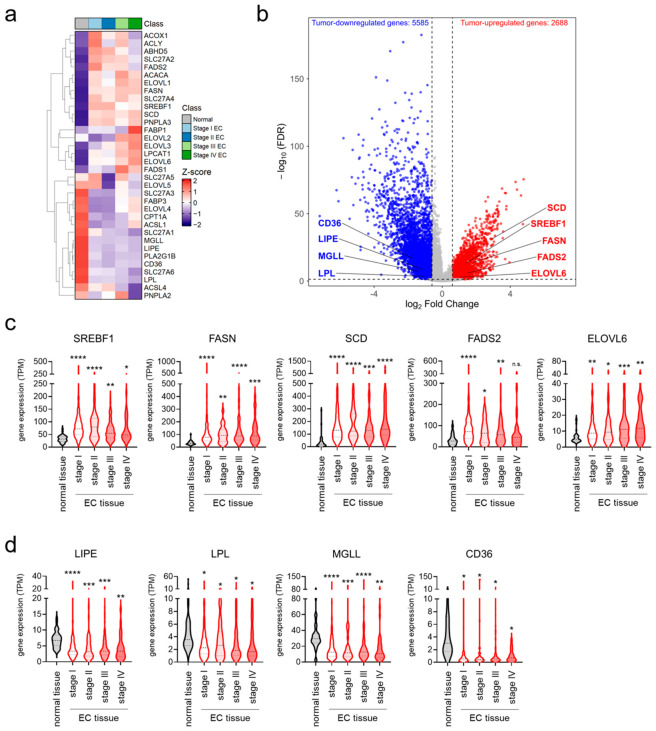
Fatty acid metabolic enzyme expression profiles are altered in EC tissue compared with normal endometrial tissue from early stages. Transcriptomic, clinical, and biospecimen data from uterine corpus endometrial carcinoma from The Cancer Genome Atlas project were used to evaluate the expression of fatty acid metabolism-related enzymes in EC tissues and normal endometrial tissues. (**a**) A heatmap illustrating the expression profiles of 34 representative fatty acid metabolism-related genes across normal endometrial and EC groups stratified by clinical stage. Gene expression values represent Z-score–normalized mean expression levels for each group, and genes were hierarchically clustered. (**b**) A volcano plot showing differentially expressed genes between normal endometrial tissues and EC tissues. Genes upregulated and downregulated in tumors are shown in red and blue, respectively, whereas nonsignificant genes (FDR > 0.05 or |log_2_ fold change| < 0.6) are shown in gray. Representative fatty acid metabolism-related genes that are consistently and significantly dysregulated from stage I onward are labeled. (**c**,**d**) Comparison of fatty acid metabolic enzyme expression levels across stages. Enzymes persistently upregulated (**c**) or downregulated (**d**) from stage I compared with normal endometrial tissue. *p* values were determined by comparing normal tissue with each group. * *p* < 0.05, ** *p* < 0.01, *** *p* < 0.001, and **** *p* < 0.0001. ns: not significant. EC, endometrial cancer; SREBF1, sterol regulatory element-binding transcription factor 1; FASN, fatty acid synthase; SCD (SCD1), stearoyl-CoA desaturase 1; FADS2, fatty acid desaturase 2; ELOVL6, elongation of very long-chain fatty acids protein 6; CD36, cluster of differentiation 36 (fatty acid translocase); LIPE, lipase E (hormone-sensitive lipase); LPL, lipoprotein lipase; MGLL, monoglyceride lipase; FDR, false discovery rate.

We previously reported that serum levels of specific FFAs fluctuate significantly due to altered expression of fatty acid metabolism-related enzymes in tumor tissues [25]. Therefore, we first evaluated the expression of various fatty acid metabolic enzymes in EC tissues using publicly available databases. Comprehensive analysis of 34 fatty acid metabolism-related enzymes identified clusters of genes that were upregulated and downregulated in EC compared with normal endometrial tissues (Figure 1a). Specifically, key enzymes involved in de novo fatty acid synthesis, including sterol regulatory element binding transcription factor 1 (SREBF1), FASN, the fatty acid desaturases SCD1 and fatty acid desaturase 2, and elongation of very long-chain fatty acids protein 6 (a fatty acid elongase), were significantly upregulated more than 1.5-fold in EC tissues (Figure 1b). Conversely, the expression levels of enzymes that liberate fatty acids from triglycerides and phospholipids (namely lipase E, lipoprotein lipase, and monoglyceride lipase), as well as CD36, a key mediator of extracellular long-chain fatty acid uptake, were markedly decreased more than 1.5-fold in tumor tissues (Figure 1b). These results suggest that metabolic alterations capable of reshaping the FFA profile within EC tumor tissues occur. Notably, these expression changes were already evident as early as stage I (Figure 1c,d) and occurred independently of histological type (Figure S1a,b), strongly suggesting that fatty acid metabolic reprogramming in EC tumor tissues occurs from the earliest stages of the disease regardless of histological type.

### 3.2. Specific Serum FFAs Are Altered in Patients with EC Independently of Clinical Stage

**Figure 2:**
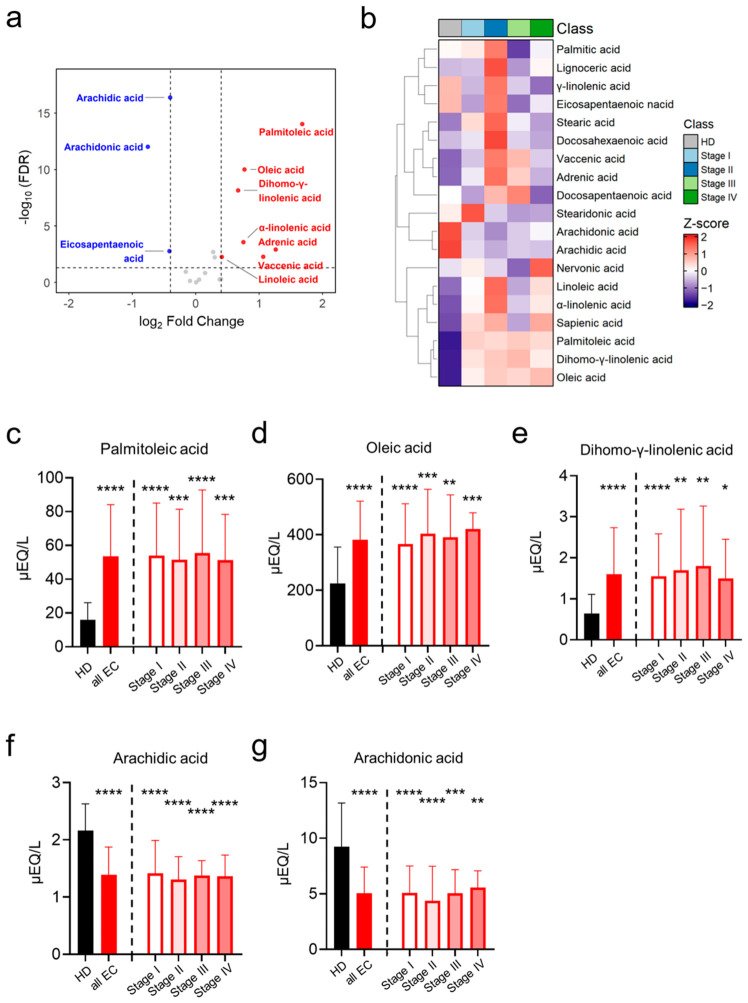
Serum FFA profiles in patients with EC differ from those in HDs. The levels of 19 FFAs (listed in Table 2) were measured by gas chromatography–mass spectrometry in serum samples from HDs (*n* = 84), patients with stage I EC (*n* = 42), patients with stage II EC (*n* = 10), patients with stage III EC (*n* = 10), and patients with stage IV EC (*n* = 10). (**a**) Volcano plot showing differences in FFA levels between HDs and all-stage EC patients. FFAs upregulated and downregulated are shown in red and blue, respectively, whereas nonsignificant FFAs (FDR > 0.05 or |log_2_ fold change| < 0.4) are shown in gray. (**b**) A heatmap showing the levels of 19 FFAs across HDs and EC groups stratified by clinical stage. (**c**–**g**) Comparison of HDs with all-stage EC or each-stage EC. Among the 19 FFAs, (**c**) palmitoleic acid, (**d**) oleic acid, (**e**) dihomo-γ-linolenic acid, (**f**) arachidic acid, and (**g**) arachidonic acid are shown because these FFAs were significantly altered from stage I. *p* values were determined by comparing HDs with each group. * *p* < 0.05, ** *p* < 0.01, *** *p* < 0.001, and **** *p* < 0.0001. FFA, free fatty acid; EC, endometrial cancer; HD, healthy donor.

Given that serum FFA levels were predicted to be altered in patients with EC, we comprehensively measured 19 FFAs by GC-MS in serum samples from 84 healthy donors and 72 patients with stage I–IV EC. Compared with healthy donors, patients with EC across all stages exhibited significantly elevated levels of palmitoleic acid, linoleic acid, α-linolenic acid, oleic acid, vaccenic acid, dihomo-γ-linolenic acid, and adrenic acid (Figure 2a and Figure S2a–f), whereas arachidic acid, arachidonic acid, and eicosapentaenoic acid were significantly decreased (Figure 2a and Figure S2g). Further stage-stratified analysis revealed that five FFAs—palmitoleic acid, oleic acid, dihomo-γ-linolenic acid, arachidic acid, and arachidonic acid—were consistently and significantly altered from stage I onward, independently of clinical stage (Figure 2b–g).

**Figure 3:**
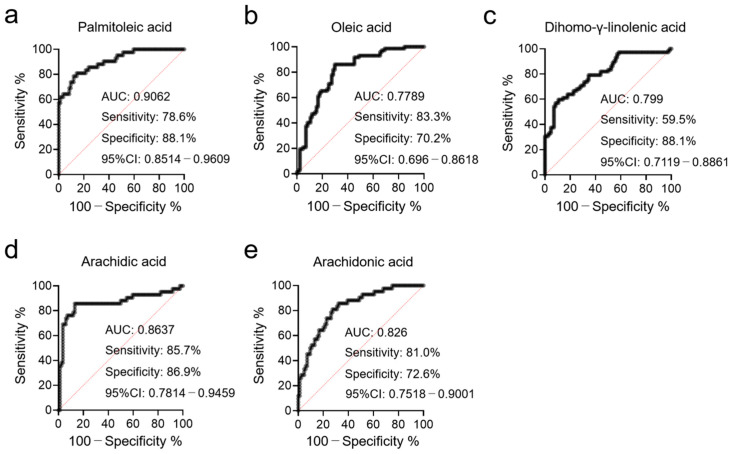
Serum free fatty acid levels are useful as diagnostic markers for stage I endometrial cancer. This figure shows receiver operating characteristic curves for detecting patients with stage I endometrial cancer using five free fatty acids identified as candidate diagnostic markers in Figure 2. The free fatty acids analyzed were (**a**) palmitoleic acid, (**b**) oleic acid, (**c**) dihomo-γ-linolenic acid, (**d**) arachidic acid, and (**e**) arachidonic acid. AUC, area under the receiver operating characteristic curve; CI, confidence interval.

These stage-independent alterations in the five FFAs warranted further investigation of their diagnostic performance. Receiver operating characteristic curve analysis comparing healthy donors and patients with stage I EC demonstrated that all five FFAs—palmitoleic acid, oleic acid, dihomo-γ-linolenic acid, arachidic acid, and arachidonic acid—exhibited high diagnostic accuracy (Figure 3a–e). These findings were consistent when the analysis was extended to include all EC stages (stage I–IV) (Figure S3a–e). Collectively, these results strongly suggest that these five FFAs enable highly accurate detection of EC, including early-stage disease.

### 3.3. A Diagnostic Model Combining Serum Palmitoleic Acid and Arachidonic Acid Is a Promising Screening Tool for EC

**Figure 4:**
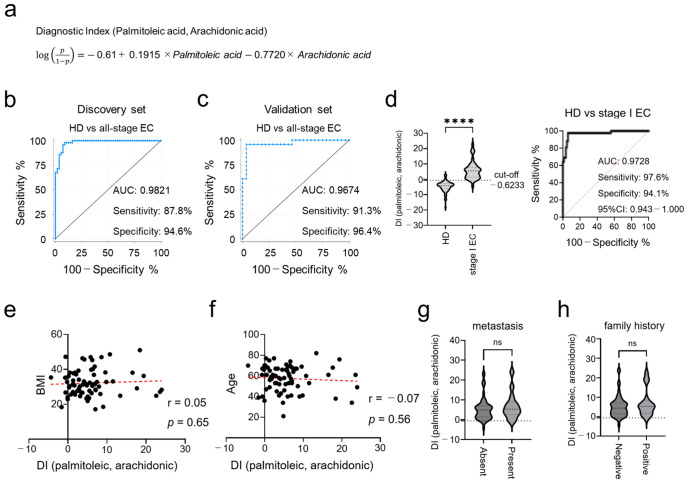
The development of an EC detection model using serum free fatty acid levels. (**a**–**c**) Diagnostic performance of the DI based on palmitoleic acid and arachidonic acid (DI [palmitoleic, arachidonic]). (**a**) The DI [palmitoleic, arachidonic] established by multi-variate logistic regression; (**b**,**c**) Receiver operating characteristic curve analysis comparing healthy donors (HDs) and patients with all-stage EC in the (**b**) discovery dataset (HDs: *n* = 56; patients with EC: *n* = 49) and (**c**) validation dataset (HDs: *n* = 28; patients with EC: *n* = 23). (**d**) The diagnostic performance of DI [palmitoleic, arachidonic] in stage I EC. Comparison of DI values between HDs and patients with stage I EC (**left panel**). Receiver operating characteristic curve analysis comparing HDs and patients with stage I EC (**right panel**). Dotted lines indicate the cut-off values. The cut-off value of DI [palmitoleic, arachidonic] was defined as the value with the highest Youden index calculated by receiver operating characteristic curve analysis. (**e**,**f**) Correlations between DI [palmitoleic, arachidonic] and (**e**) BMI and (**f**) age. (**g**,**h**) Comparison of DI [palmitoleic, arachidonic] between presence or absence of metastasis (**g**) or family history of cancer (**h**). **** *p* < 0.0001. ns: not significant. HD, healthy donor; EC, endometrial cancer; AUC, area under the receiver operating characteristic curve; DI, diagnostic index; BMI, body mass index.

Having identified FFAs capable of detecting EC across all stages, we next assessed whether combining specific fatty acids could further improve diagnostic accuracy. To develop a screening tool applicable to a broad population, we first aimed to establish a diagnostic model capable of reliably discriminating patients with EC (stage I–IV) from healthy donors. Using a discovery dataset (healthy donors: *n* = 56; patients with EC: *n* = 49), candidate FFAs were selected and evaluated as described in the Methods Section. This analysis identified the combination of palmitoleic acid and arachidonic acid as the optimal diagnostic model, expressed as DI = −0.61 + 0.1915 × (palmitoleic acid) − 0.7720 × (arachidonic acid) (Figure 4a). Receiver operating characteristic curve analysis of this model yielded an AUC of 0.9821, with a sensitivity of 87.8% and specificity of 94.6% (Figure 4b). Validation in an independent dataset (healthy donors: *n* = 28; patients with EC: *n* = 23) confirmed the model’s robustness, with an AUC of 0.9674, sensitivity of 91.3%, and specificity of 96.4% (Figure 4c). Notably, when the analysis was restricted to stage I EC, the model clearly distinguished healthy donors from patients with EC, retaining strong performance with an AUC of 0.9728, sensitivity of 97.6%, and specificity of 94.1% (Figure 4d), confirming its utility for early-stage detection.

Because EC frequently occurs in patients with obesity or advanced age, it was critical to determine whether FFA levels are influenced by these factors. Accordingly, we evaluated the relationship between the diagnostic model and BMI and age. The diagnostic model was found to be independent of these factors (Figure 4e,f). Furthermore, the diagnostic index was not influenced by the presence or absence of metastasis (Figure 4g) or family history of cancer (Figure 4h), suggesting that the model reflects a metabolic alteration intrinsic to EC tumor tissues rather than factors associated with disease progression or hereditary predisposition. Taken together, these findings further support the utility of the palmitoleic acid and arachidonic acid diagnostic model as a robust and broadly applicable screening tool for EC.

## 4. Discussion

In developed countries, the incidence of EC has been increasing, driven by rising rates of obesity and metabolic syndrome [1,2,3,4]; however, when diagnosed early, most cases are curable with favorable outcomes [7,8]. Therefore, the establishment of noninvasive screening strategies capable of detecting EC at an early stage is urgently needed. Recent research has focused on various metabolic alterations in cancer cells, which are being used for patient stratification. In this study, we demonstrated that the expression of various fatty acid metabolic enzymes is altered from early stages in EC tissue compared with normal endometrial tissue. Furthermore, compared with healthy individuals, serum levels of palmitoleic acid and oleic acid were significantly elevated in patients with EC, whereas arachidic acid and arachidonic acid were significantly decreased, enabling clear discrimination between the two groups. Using statistical methods, we constructed a diagnostic model combining palmitoleic acid and arachidonic acid. This diagnostic model demonstrated high diagnostic performance regardless of clinical stage and was confirmed to be independent of known risk factors, including BMI, aging, and family history. These findings suggest that a noninvasive approach based on serum FFA profiling has the potential to serve as a screening strategy for EC, addressing an important unmet clinical need.

In this study, we found that the expression of multiple enzyme families involved in fatty acid metabolism is coordinately altered in EC tissue compared with normal endometrial tissue. Specifically, increased expression was observed in SREBF1 and FASN, which are responsible for de novo fatty acid synthesis, as well as SCD1, the rate-limiting enzyme for monounsaturated fatty acid biosynthesis, consistent with our previous reports [25,26]. By contrast, the expression of fatty acid release enzymes, including lipoprotein lipase, lipase E, and monoglyceride lipase [31], as well as CD36, which regulates the uptake of extracellular long-chain fatty acids [32], was significantly decreased. This suggests that tumor cells remodel their lipid microenvironment by suppressing dependence on exogenous fatty acid uptake while enhancing endogenous fatty acid synthesis and modification.

This fatty acid metabolic reprogramming in EC is likely closely associated with obesity, a major risk factor for EC, and the accompanying activation of insulin signaling pathways [33,34]. Obesity-associated insulin signaling activates SREBF1, a master transcriptional regulator that induces the expression of downstream lipogenic enzymes, including FASN and SCD1 [35,36]. The increased expression of these enzymes provides tumor cells with the membrane lipids required for rapid proliferation and likely contributes to the formation of a tumor microenvironment favorable for tumor cell survival through SCD1-mediated production of monounsaturated fatty acids, which increases cell membrane fluidity and reduces endoplasmic reticulum stress [37]. Interestingly, these alterations in fatty acid metabolic enzyme expression were already present from stage I, indicating that fatty acid metabolic reprogramming is not a secondary phenomenon of late-stage tumor progression but rather a fundamental metabolic feature involved from the earliest stages of EC development. This provides a theoretical basis for the detection of serum FFA changes from the early stages of disease.

In this study, we identified five FFAs that were significantly altered in the serum of patients with EC from stage I. Specifically, palmitoleic acid, oleic acid and dihomo-γ-linolenic acid were elevated, whereas arachidic acid and arachidonic acid were decreased. Among these, palmitoleic acid and oleic acid were also elevated in our previous studies of ovarian and cervical cancers [25,26]. Both fatty acids are products of SCD1, whose expression was upregulated in EC tissue in the present study. This consistency across three gynecological cancer types strongly supports the concept that SCD1-driven production of monounsaturated fatty acids is a common metabolic feature in cancers with fatty acid metabolic reprogramming, and that the resulting serum elevations of palmitoleic acid and oleic acid may serve as broadly applicable indicators of cancer-associated fatty acid metabolic reprogramming. Indeed, elevated serum levels of palmitoleic acid and oleic acid have also been reported in breast cancer [38], further supporting their potential as broadly applicable cancer biomarkers.

Conversely, some of the altered FFAs appear to be more specific to EC. Arachidonic acid was elevated in cervical cancer but decreased in EC, suggesting that the direction of change in specific FFAs may reflect cancer type-specific metabolic characteristics. Arachidonic acid is a precursor of prostaglandins and leukotrienes, which play critical roles in inflammatory responses and tumor microenvironment regulation [39]. In EC, reduced serum arachidonic acid levels could potentially reflect increased consumption by cyclooxygenase-2, which is frequently overexpressed in EC tissue and converts arachidonic acid to prostaglandin E2, a mediator known to promote tumor proliferation, angiogenesis, and immune evasion [39,40]. In addition, decreased arachidic acid levels in EC serum are consistent with our previous findings in ovarian cancer and may be attributable to substrate competition with SCD1 because both oleic acid and arachidic acid are derived from stearic acid [25]. Beyond their roles within tumor cells, these fatty acid alterations may also act on non-tumor components of the tumor microenvironment. In particular, the reduced arachidonic acid levels observed here, together with enhanced cyclooxygenase-2–mediated conversion of arachidonic acid to prostaglandin E2, can modulate the function of tumor-infiltrating immune cells, including T cells and macrophages, and influence stromal cell behavior, potentially fostering an immunosuppressive, pro-tumorigenic niche [39,40]. How EC-associated shifts in fatty acid composition act on these non-tumor cells was not directly examined in the present study and represents an important direction for future mechanistic investigation.

The identification of both cross-cancer and cancer type-specific FFA alterations has important implications for diagnostic strategy. While shared FFAs such as palmitoleic acid and oleic acid may be useful for initial cancer screening regardless of cancer type, the inclusion of cancer type-specific FFAs in diagnostic models enables differentiation among cancer types. In the present study, the optimal diagnostic model for EC was based on a combination of palmitoleic acid and arachidonic acid, which differs from the oleic acid and arachidic acid combination established for ovarian cancer [25] and the stearic acid and dihomo-γ-linolenic acid combination for cervical cancer [26]. This suggests that cancer type-specific serum FFA profiles can be leveraged to develop targeted diagnostic models for individual cancer types within the broader context of fatty acid metabolic reprogramming.

Current EC diagnosis mainly targets symptomatic patients, and no established screening method exists for detecting asymptomatic early-stage disease. Transvaginal ultrasonography, though noninvasive, has limited specificity because of overlapping endometrial thickness measurements between benign and malignant conditions, while endometrial biopsy, the diagnostic gold standard, is invasive and associated with procedural failure rates that limit its clinical applicability [6,13]. Furthermore, tumor markers such as CA125, CEA, and CA19-9, which are often routinely measured in gynecological outpatient settings, have been shown to be inadequate as screening tools for EC. These limitations underscore the clinical value of noninvasive and highly accurate novel biomarkers.

The diagnostic model combining palmitoleic acid and arachidonic acid established in this study addresses these unmet needs. In the independent validation dataset, this model achieved an AUC of 0.9674, with a sensitivity of 91.3% and specificity of 96.4%. Notably, even in the analysis limited to stage I EC, the model maintained an AUC of 0.9728, with a sensitivity of 97.6% and specificity of 94.1%, demonstrating excellent performance in detecting early-stage disease. This noninvasive approach, requiring only a simple blood test, enhances patient acceptability and facilitates application to large-scale screening.

Another important feature of this diagnostic model is its independence from BMI and age. Given that EC incidence increases with obesity and aging, independence from these factors ensures consistent diagnostic performance across a broad target population. Furthermore, given that the alterations in fatty acid metabolic enzyme expression were independent of histological type, this model is likely broadly applicable regardless of tumor histology. Together with its independence from family history of cancer, these characteristics suggest that the diagnostic model is a useful tool that can complement existing diagnostic methods in detecting EC across diverse patient backgrounds and tumor characteristics.

This study has several limitations. First, we compared only healthy controls and patients with EC; we did not include patients with benign gynecological diseases, such as uterine fibroids, adenomyosis, and endometriosis, or systemic diseases that affect lipid metabolism, such as diabetes mellitus and hyper-lipidemia. These conditions may also influence serum FFA composition and could potentially lead to false positive results. Consequently, the specificity of our diagnostic model and its false positive rate in a screening context cannot be determined from the present case–control design. Future studies including these disease control groups are necessary to more precisely evaluate the specificity of our diagnostic model before clinical translation. Second, our analysis of expression changes in EC tissue was limited to the transcriptomic level; whether these changes are also manifested at the protein or functional level was not examined, and validation in independent EC tissue is required. Third, the precise biological mechanisms underlying changes in serum FFA composition have not been fully elucidated. Although we demonstrated that fatty acid metabolic enzyme expression is altered in EC tissue, the mechanisms linking tissue-level fatty acid metabolic reprogramming to systemic alterations in serum FFA composition remain to be fully characterized and likely involve complex pathways beyond simple tumor-derived release. In particular, the cross-sectional design of this study makes it difficult to determine the extent to which these serum changes are attributable to the tumor versus host systemic metabolism. Moreover, as only preoperative samples were analyzed, whether the FFA alterations are reversible upon tumor removal remains to be determined in future longitudinal studies. Thus, further mechanistic investigation is warranted to strengthen the biological basis of this diagnostic approach. Fourth, the serum FFA cohort was not stratified by histological subtype; so, the performance of the diagnostic model in aggressive non-endometrioid histologies such as serous and clear cell carcinoma remains to be confirmed. Despite these limitations, our study is significant in being the first to demonstrate that serum FFA profiling may be useful for early diagnosis of EC, providing a foundation for future large-scale validation studies and clinical translation.

This study demonstrated that fatty acid metabolic reprogramming in EC tissue occurs from stage I and that the associated changes in serum FFA composition can be utilized for early diagnosis. The diagnostic model combining palmitoleic acid and arachidonic acid showed high diagnostic accuracy and was independent of BMI, age, and histological type, suggesting its potential as a noninvasive screening tool that could complement the limitations of existing diagnostic methods. Together with our previous findings in ovarian and cervical cancers, these results indicate that serum FFA profiling represents a promising diagnostic strategy across gynecological malignancies. Future large-scale prospective multicenter studies are warranted to validate the clinical utility of this diagnostic model and are expected to contribute to the early detection of EC and improvement in patient outcomes. Incorporating conventional tumor markers into such studies will further allow for direct comparison with serum FFA profiles and clarify their potential complementary diagnostic value.

## 5. Conclusions

Serum FFA profiling offers a noninvasive, blood-based approach to EC screening that is independent of BMI, age, family history of cancer, and tumor characteristics including stage, metastatic status, and histological type, addressing key limitations of current diagnostic methods. The diagnostic model combining palmitoleic acid and arachidonic acid may serve as a practical screening tool applicable to a broad population, including younger women in whom EC incidence is increasingly recognized, demonstrating robust performance even for early-stage disease detection.

## Figures and Tables

**Table 1:** Characteristics of participants whose serum free fatty acid concentrations were measured.

Characteristics	Healthy Donors (*n* = 84)	Endometrial Cancer Patients (*n* = 72)
**Age**		
median (range)	61.0 (37–83)	59.5 (21–82)
**Metastasis**		
absent/present		12/60
**BMI**		
median (range)		31.2 (17.1–51.0)
**Family history of cancer**		
negative/positive		56/16
**Pathological stage**		
I (IA/IB)		42 (32/10)
II		10
III (IIIA/IIIB/IIIC)		10 (2/1/7)
IV (IVA/IVB)		10 (5/5)

BMI, body mass index.

**Table 2:** Gas chromatography–mass spectrometry analyses of serum free fatty acids.

Compound	Target Ion *m*/*z*	Rt (min)	LOD (ng/20 μL)	LOQ (ng/20 μL)
Sapienic acid	311.2	5.720	0.13	0.32
Palmitoleic acid	311.2	5.750	0.13	0.32
Palmitic acid	313.2	5.833	0.09	0.22
γ-linolenic acid	335.2	6.569	1.89	4.96
Stearidonic acid	333.2	6.611	0.75	1.75
Linoleic acid	337.2	6.652	0.04	0.08
Oleic acid	339.2	6.669	0.09	0.24
α-linolenic acid	335.2	6.698	0.19	0.48
Vaccenic acid	339.2	6.703	0.09	0.24
Stearic acid	341.2	6.790	0.08	0.21
Arachidonic acid	361.2	7.540	2.58	6.58
Eicosapentaenoic acid	359.2	7.595	9.65	21.31
Dihomo-γ-linolenic acid	363.2	7.657	0.15	0.30
Arachidic acid	369.2	7.917	0.12	0.31
Docosahexaenoic acid	385.25	8.798	0.91	2.25
Adrenic acid	389.25	8.842	4.35	8.93
Docosapentaenoic acid	387.25	8.915	0.43	1.02
Nervonic acid	423.3	10.745	0.03	0.07
Lignoceric acid	425.3	10.938	0.01	0.02
Margaric acid	327.2	6.293	I.S.	I.S.

Rt, retention time; LOD, limit of detection; LOQ, limit of quantification; I.S., internal standard.

## Data Availability

The authors declare that all other data supporting the findings of this study are available within the article and its Supplementary Materials, and from the corresponding author upon reasonable request.

## Supplementary Materials

The following supporting information can be downloaded at: https://www.mdpi.com/article/10.3390/cancers18132133/s1, Figure S1: Fatty acid metabolic enzyme expression profiles are altered in EC tissue independently of histological type (related to Figure 1); Figure S2: Serum levels of seven free fatty acids are altered in EC but do not show consistent stage-independent changes (related to Figure 2); Figure S3: Serum free fatty acid levels are useful as diagnostic markers for stage I–IV endometrial cancer.

## Abbreviations

The following abbreviations are used in this manuscript:

EC	endometrial cancer
FFA	free fatty acid
BMI	body mass index
SCD1	stearoyl-CoA desaturase 1
FASN	fatty acid synthase
FIGO	International Federation of Gynecology and Obstetrics
GC-MS	gas chromatography–mass spectrometry
FDR	false discovery rate
AUC	area under the receiver operating characteristic curve
DI	diagnostic index
SREBF1	sterol regulatory element binding transcription factor 1
