# Supplementary material for: Serum Palmitoleic Acid and Arachidonic Acid as a Noninvasive Screening Tool for Endometrial Cancer
Source: Cancers (Basel). 2026 Jul 1;18(13):2133. doi: 10.3390/cancers18132133 (PMC13360203; doi:10.3390/cancers18132133)
Supplement: Supplementary file 1 [file cancers-18-02133-s001.zip › cancers-4390158-supplementary.pdf]

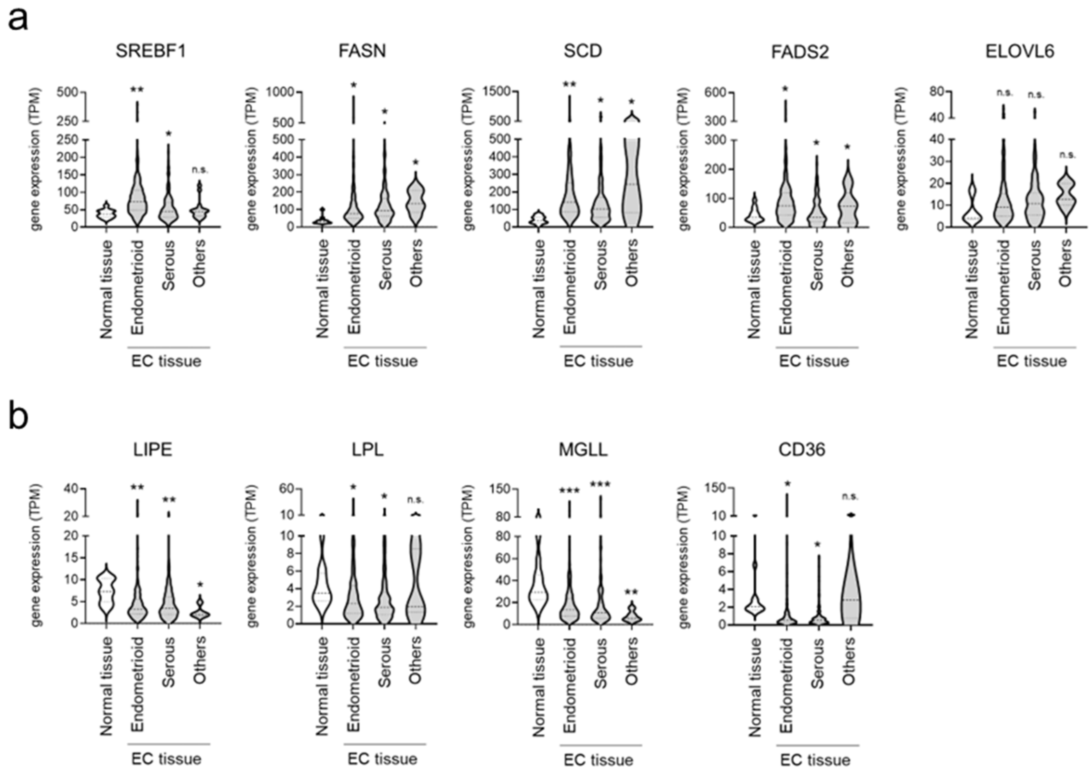

Supplemental Figure S1. Fatty acid metabolic enzyme expression profiles are altered in EC tissue independently of histological type (related to Figure 1). (a, b) Comparison of fatty acid metabolic enzyme expression levels across histological types. Enzymes consistently upregulated **(a)** or downregulated **(b)** in EC tissues compared with normal endometrial tissue, regardless of histological type. p values were determined by comparing normal tissue with each group. $*p < 0.05$, $**p < 0.01$, $***p < 0.001$. ns: not significant. EC, endometrial cancer; SREBF1, sterol regulatory element-binding transcription factor 1; FASN, fatty acid synthase; SCD (SCD1), stearoyl-CoA desaturase 1; FADS2, fatty acid desaturase 2; ELOVL6, elongation of very long-chain fatty acids protein 6; CD36, cluster of differentiation 36 (fatty acid translocase); LIPE, lipase E (hormone-sensitive lipase); LPL, lipoprotein lipase; MGLL, monoglyceride lipase; FDR, false discovery rate.

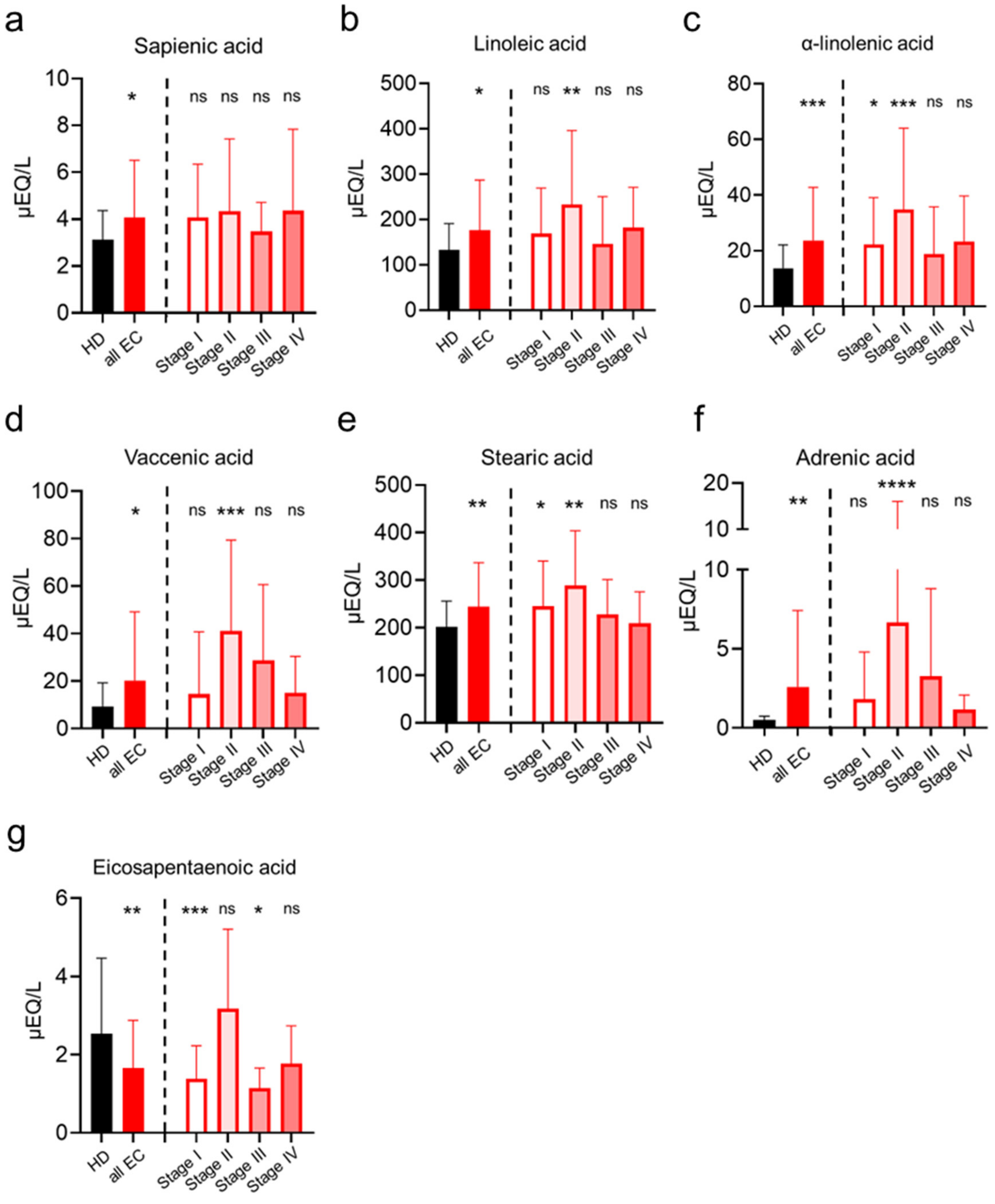

Supplemental Figure S2. Serum levels of seven free fatty acids are altered in EC but do not show consistent stage-independent changes (related to Figure 2). (a–g) Comparison of serum free fatty acid levels between HDs and the all-stage EC or each-stage EC groups. Data are presented for **(a)** sapienic acid, **(b)** linoleic acid, **(c)** α-linolenic acid, **(d)** vaccenic acid, **(e)** stearic acid, **(f)** adrenic acid, and **(g)** eicosapentaenoic acid. p values were determined by comparing HDs with each group. *$p < 0.05$, **$p < 0.01$, ***$p < 0.001$, ****$p < 0.0001$. HD, healthy donor; EC, endometrial cancer; ns, not significant.

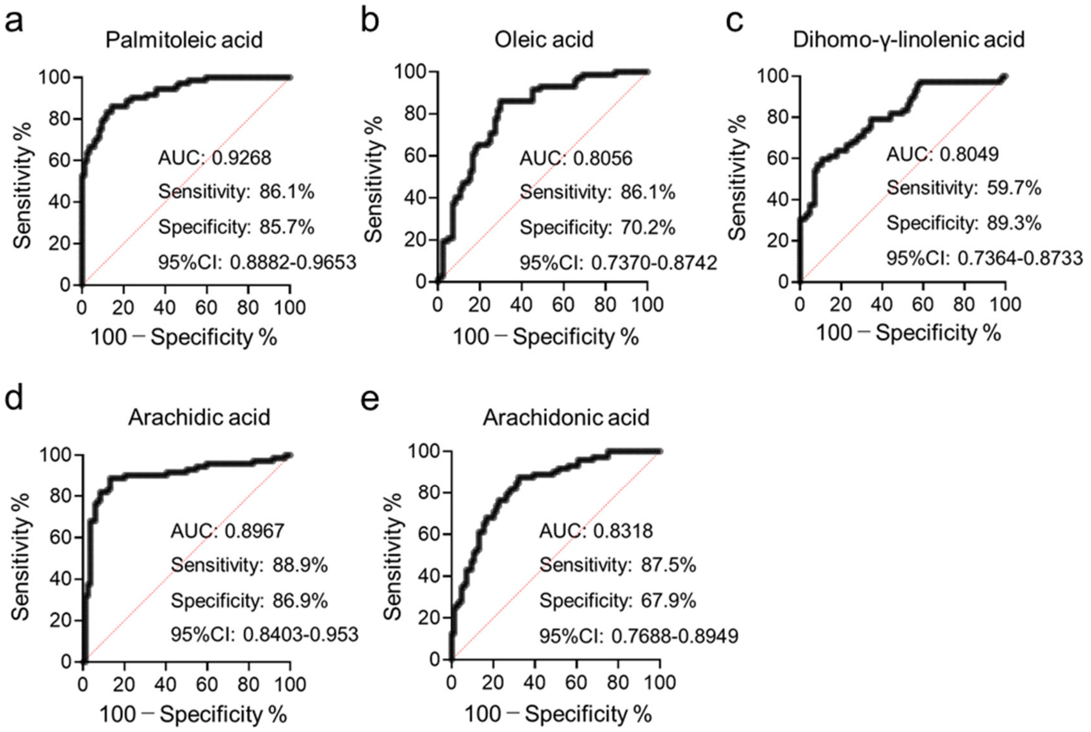

Supplemental Figure S3. Serum free fatty acid levels are useful as diagnostic markers for stage I–IV endometrial cancer. (a–e) Receiver operating characteristic curves for detecting patients with endometrial cancer across all stages (I–IV) using five free fatty acids identified as candidate diagnostic markers in Figure 2. The free fatty acids analyzed were **(a)** palmitoleic acid, **(b)** oleic acid, **(c)** dihomo-γ-linolenic acid, **(d)** arachidic acid, and **(e)** arachidonic acid. AUC, area under the receiver operating characteristic curve; CI, confidence interval.
